# The Impact of Filter Settings on Morphology of Unipolar Fibrillation Potentials

**DOI:** 10.1007/s12265-020-10011-w

**Published:** 2020-05-14

**Authors:** Roeliene Starreveld, Paul Knops, Maarten Roos-Serote, Charles Kik, Ad J. J. C. Bogers, Bianca J. J. M. Brundel, Natasja M. S. de Groot

**Affiliations:** 1 Department of Cardiology, Erasmus Medical Center, Doctor Molewaterplein 40, 3015 GD Rotterdam, the Netherlands; 2 Department of Cardiothoracic Surgery, Erasmus Medical Center, Rotterdam, the Netherlands; 3 Department of Physiology, Amsterdam UMC, Vrije Universiteit Amsterdam, Amsterdam Cardiovascular Sciences, Amsterdam, the Netherlands

**Keywords:** Atrial fibrillation, High-resolution epicardial mapping, Electrogram morphology, Cardiac electrophysiology, Filter settings

## Abstract

Using unipolar atrial electrogram morphology as guidance for ablative therapy is regaining interest. Although standardly used in clinical practice during ablative therapy, the impact of filter settings on morphology of unipolar AF potentials is unknown. Thirty different filters were applied to 2,557,045 high-resolution epicardial AF potentials recorded from ten patients. Deflections with slope ≤ − 0.05 mV/ms and amplitude ≥ 0.3 mV were marked. *High-pass* filtering decreased the number of detected potentials, deflection amplitude, and percentage of fractionated potentials (≥ 2 deflections) as well as fractionation delay time (FDT) and increased percentage of single potentials. *Low-pass* filtering decreased the number of potentials, percentage of fractionated potentials, whereas deflection amplitude, percentage of single potentials, and FDT increased. *Notch* filtering (50 Hz) decreased the number of potentials and deflection amplitude, whereas the percentage of complex fractionated potentials (≥ 3 deflections) increased. Filtering significantly impacted morphology of unipolar fibrillation potentials, becoming a potential source of error in identification of ablative targets.

**Electronic supplementary material:**

The online version of this article (10.1007/s12265-020-10011-w) contains supplementary material, which is available to authorized users.

**Graphical Abstract:**
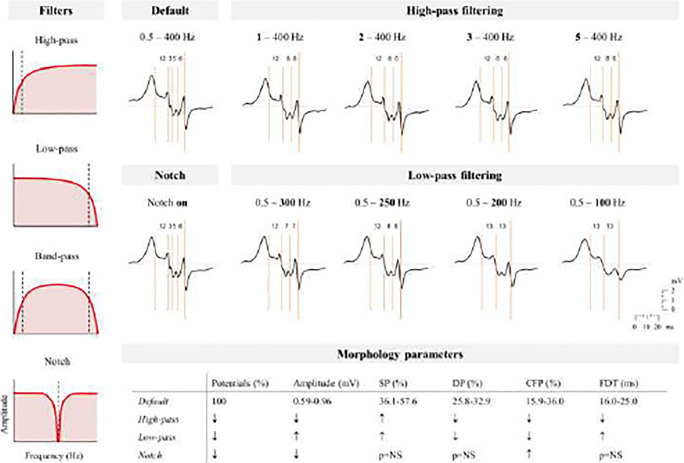
Impact of filtering on morphology of unipolar AF potentials. High-pass, low-pass and notch filters were applied to 2,557,045 high-resolution epicardial AF potentials recorded from ten patients. Filtering significantly impacted AF potential morphology, i.e., number of detected potentials, peak-to-peak amplitude, number of deflections, and fractionation delay time. CFP, complex fractionated potential (≥ 3 deflections); DP, double potential (two deflections); FDT, fractionation delay time; SP, single potential (one deflection).

## Introduction

Although insight into the pathophysiologic basis of fractionated atrial electrograms has increased in the past years, its “fact or artifact” remains a topic of debate. Extracellular electrograms—recorded directly from the heart—are generated by depolarization of cardiomyocytes, so signal morphology could provide information about the electrophysiological characteristics of the underlying myocardium. [1] Atrial potentials consisting of multiple components (i.e., fractionated) have been linked to abnormal conduction and arrhythmogenicity in patients with atrial fibrillation (AF) [1, 2], which led to targeted ablation of such fractioned potentials. However, the link between morphology of atrial potentials and pathology has proven to be anything but straightforward, considering that multiple physiological mechanisms and measurement properties, such as filtering, can cause fractionated potentials as well [3, 4]. In the 1980s, Waxman and Sung discovered the phenomenon of frequency-dependent fractionation in human bipolar ventricular electrograms [5]. Klitzner and Stevenson showed that increasing the high-pass filter frequency above 10 Hz decreased potential duration and amplitude, whereas low-pass filtering altered potential amplitude slightly if decreased up to 100 Hz [6]. Such low- and high-pass filters are commonly used in clinical practice, as well as utilization of a 50 Hz (or 60 Hz) notch filter to suppress power-line interference.

Despite the clinical failure of targeting complex fractionated atrial electrograms (CFAEs) as stand-alone therapy [7–9], using atrial electrogram morphology as guidance for ablative therapy is regaining interest [10]. Up till recently, most clinically used ablative systems preferred bipolar above unipolar measurements, given its ability to reduce far-field potentials [11]. However, bipolar signals fail to represent incoherent waves during AF, which is why currently emerging innovative ablative systems, such as RhythmView™ and AcQMap®, prefer unipolar electrograms to identify local activation [12, 13]. As the study of van der Does et al. demonstrated electrogram morphology at the epi- and endocardium to be comparable [14], epicardial electrograms are suitable to investigate signal morphology, particularly as direct contact between the electrode and atrial tissue can be assured.

To our knowledge, the impact of filtering on *unipolar fibrillation* potentials has never been investigated in humans. This study therefore aims to elucidate the consequences of high-pass, low-pass, and notch filtering on unipolar fibrillation potentials in AF patients.

## Methods

### Study Population

The study population consisted of ten adult patients with a history of paroxysmal or persistent AF undergoing elective open-heart mitral valve surgery in the Erasmus Medical Center Rotterdam. This study was approved by the institutional medical ethical committee (MEC 2010–054/MEC 2014-393) [15, 16]. Written informed consent was obtained from all patients. Patient characteristics (e.g., age, medical history, date of AF diagnosis) were obtained from the patient’s file.

### Mapping Procedure

**Fig. 1:**
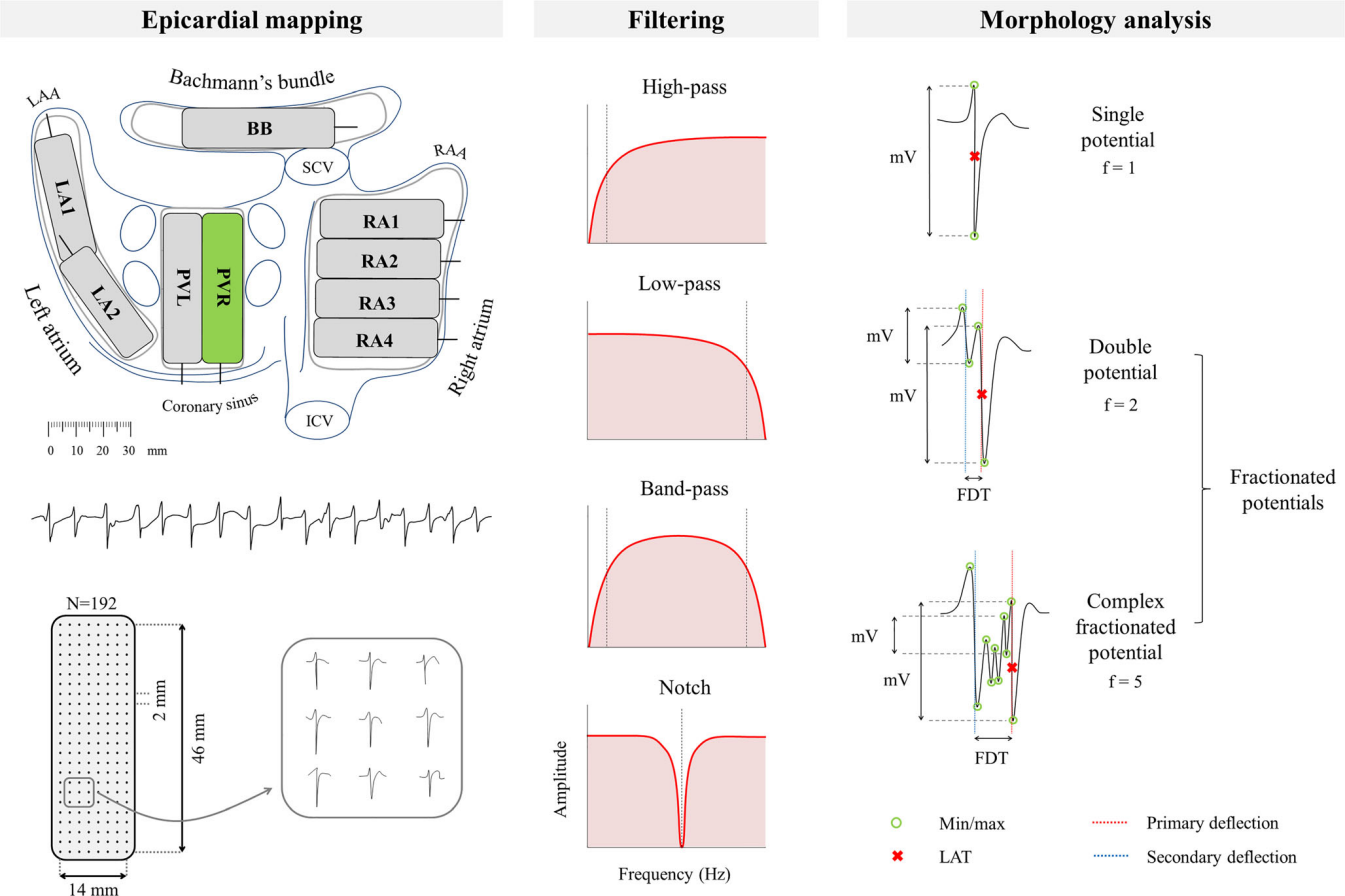
Schematic representation of mapping analysis. *Left*: the right pulmonary vein (PVR) area is mapped from the sinus transversus fold along the borders of the PVR down towards the atrioventricular groove. Using a 192-electrode array 10 s of AF is recorded. *Middle*: simplistic illustration of different filtering modes. *Right*: morphology analysis of all fibrillation potentials. The peak-to-peak amplitude of each deflection (mV), fractionation (f, number of deflections), and fractionation delay time (FDT) is derived. Fibrillation potentials were classified as either single potential (*f* = 1), double potential (*f* = 2), or complex fractionated potential (*f* ≥ 3). The steepest deflection of a potential is classified as the primary deflection (and marked as the local activation time), whereas additional deflections are classified as secondary deflections. BB, Bachmann’s bundle; ICV, inferior caval vein; LA, left atrium; LAA, left atrial appendage; LAT, local activation time; PVL, left pulmonary vein; RA, right atrium; RAA, right atrial appendage; SCV, superior caval vein

Epicardial high-resolution mapping was performed prior to commencement to extra-corporal circulation, as previously described in detail [17–19]. A temporal bipolar epicardial pacemaker wire attached to the RA free wall served as a reference electrode. A steel wire fixed to the subcutaneous tissue of the thoracic cavity was used as an indifferent electrode. Epicardial mapping was performed with a 192-electrode array (electrode diameter 0.45 mm, interelectrode distances 2.0 mm). The right pulmonary vein (PV) area was mapped from the sinus transversus fold along the borders of the right pulmonary vein down towards the atrioventricular groove (as illustrated in the left panel of Fig. 1). Ten seconds of AF were recorded, including a surface ECG lead, a calibration signal of 2 mV and 1000 ms, a bipolar reference electrogram, and all unipolar epicardial electrograms. Data was stored on a hard disk after amplification (gain 1000), filtering (band-pass 0.5–400 Hz), sampling (1 kHz), and analog to digital conversion (16 bits).

### Filter Settings

The impact of additional high-pass, low-pass, and notch filtering (i.e., narrow band-stop filter), as illustrated in the middle panel of Fig. 1, was investigated by changing filter settings one at a time, while keeping the others at default:

- High-pass filter: 0.5 (default), 1, 2, 3, 5, 10, 20, 30, 40, 50, 60, 70, 75, 80, 90, 100 Hz
- Low-pass filter: 400 (default), 300, 250, 200, 150, 100, 75, 60, 50, 40, 30, 20, 10 Hz
- Notch filter at 50 Hz: off (default) and on

Settings were based on frequently used filter options within clinical mapping systems. Signals were zero-phase filtered with IIR Butterworth low- and high-pass filters (2nd-order: 12 dB/octave roll-off) and/or IIR notch filter with a quality factor of 30. Bode plots of the three filters are illustrated in ESM 1.

### Data Analysis

Electrogram morphology was semi-automatically analyzed in custom-made Python 3.6 software. Deflections of atrial potentials were automatically marked if the slope was ≤ − 0.05 mV/ms and the amplitude ≥ 0.3 mV; the refractory period was set to 40 ms [20]. The steepest negative deflection of a potential was classified as the primary deflection and marked as the local activation time (LAT), whereas—in case of a fractionated potential—additional deflections were classified as secondary deflections. Electrograms with injury potentials and artifacts were excluded from analysis by manual assessment and consensus of two independent investigators.

For each different filter setting, peak-to-peak amplitude (voltage), fractionation (*f*, number of deflections), and fractionation delay time (FDT, time interval between first and last deflection) of atrial potentials were analyzed. Peak-to-peak amplitude was analyzed for all deflections, as well as for primary and secondary deflections separately. FDT was only derived for fractionated potentials (*f* ≥ 2). For each morphology parameter median values of all atrial potentials within the 192-array were derived and compared between filter settings. In addition, each fibrillation potential was classified as either single potential (SP, *f* = 1), double potential (DP, *f* = 2), or complex fractionated potential (CFP, *f* ≥ 3). Figure 1 (right panel) illustrates derivation of morphology parameters and classification of fibrillation potentials.

### Statistical Analysis

The impact of filtering on characteristics of unipolar fibrillation potentials, including amplitude, fractionation, and FDT, was analyzed using linear mixed-effect models, while accounting for clustered data within a patient. Analyses were done for the three different types of filtering separately: low-pass, high-pass, and notch filtering. The basic model only included a random intercept and presumed no relation between filtering and morphology characteristics. Based on the Akaike Information Criterion it was checked whether addition of a random slope improved the model. To model any non-linearity two splines were used. Residual plots were reviewed and log or square root transformed data was used if deviations from normality were observed. Statistical significance was tested using the likelihood ratio test. A *p* value < 0.05 was considered statistically significant. All statistical analyses were performed using R Statistical Software (RStudio, Inc., Boston, MA; version 1.0.153).

## Results

Patient characteristics (*n* = 10) are shown in Table 1. All patients had a history of AF (paroxysmal *n* = 4, persistent *n* = 6) and age ranged from 56 to 77 years. In total, 3000 s of AF recordings were analyzed, consisting of 2,557,045 fibrillation potentials.

**Table 1:** Patient characteristics. *AF* atrial fibrillation, *BMI* body mass index, *F* female, *IHD* ischemic heart disease, *M* male, *MVD* mitral valve disease

Study ID	Underlying heart disease	Age (years)	Gender	BMI	Type of AF	Time since AF diagnosis (years)
1	MVD	70	M	25.3	Paroxysmal	5.61
2	MVD + IHD	75	M	32.3	Persistent	2.07
3	MVD + IHD	67	M	21.8	Persistent	0.25
4	MVD	65	M	25.6	Persistent	20.33
5	MVD	77	F	26.7	Persistent	0.72
6	MVD	66	M	23.2	Persistent	1.13
7	MVD	56	M	26.4	Persistent	0.61
8	MVD + IHD	70	M	24.2	Paroxysmal	0.06
9	MVD	64	F	34.6	Paroxysmal	0.59
10	MVD	74	M	22.1	Paroxysmal	0.28

**Fig. 2:**
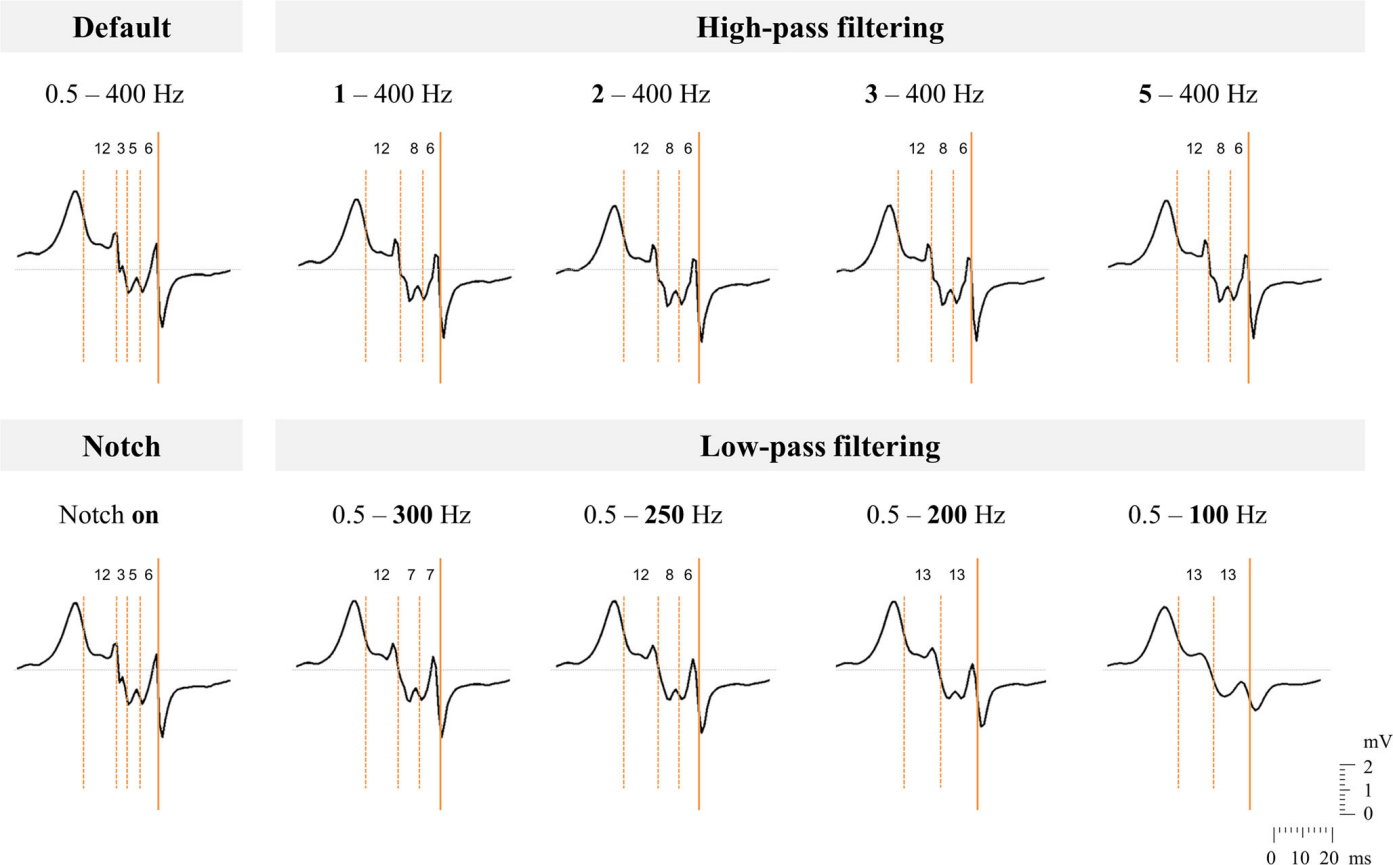
Illustration of the impact of frequently used filter settings, i.e., high-pass, low-pass, and notch filtering, on morphology of one fractionated unipolar fibrillation potential. Detected deflections are marked with orange vertical lines, a solid line representing the primary deflection and dashed lines the secondary deflections. The corresponding time interval between adjacent deflections is given (in ms)

The impact of frequently used high-pass, low-pass, and notch filter settings on morphology of one example of a fractionated fibrillation potential is illustrated in Fig. 2. General consequences of all filter settings are discussed in the sections below.

### Impact of High-Pass Filtering on Morphology of Fibrillation Potentials

**Fig. 3:**
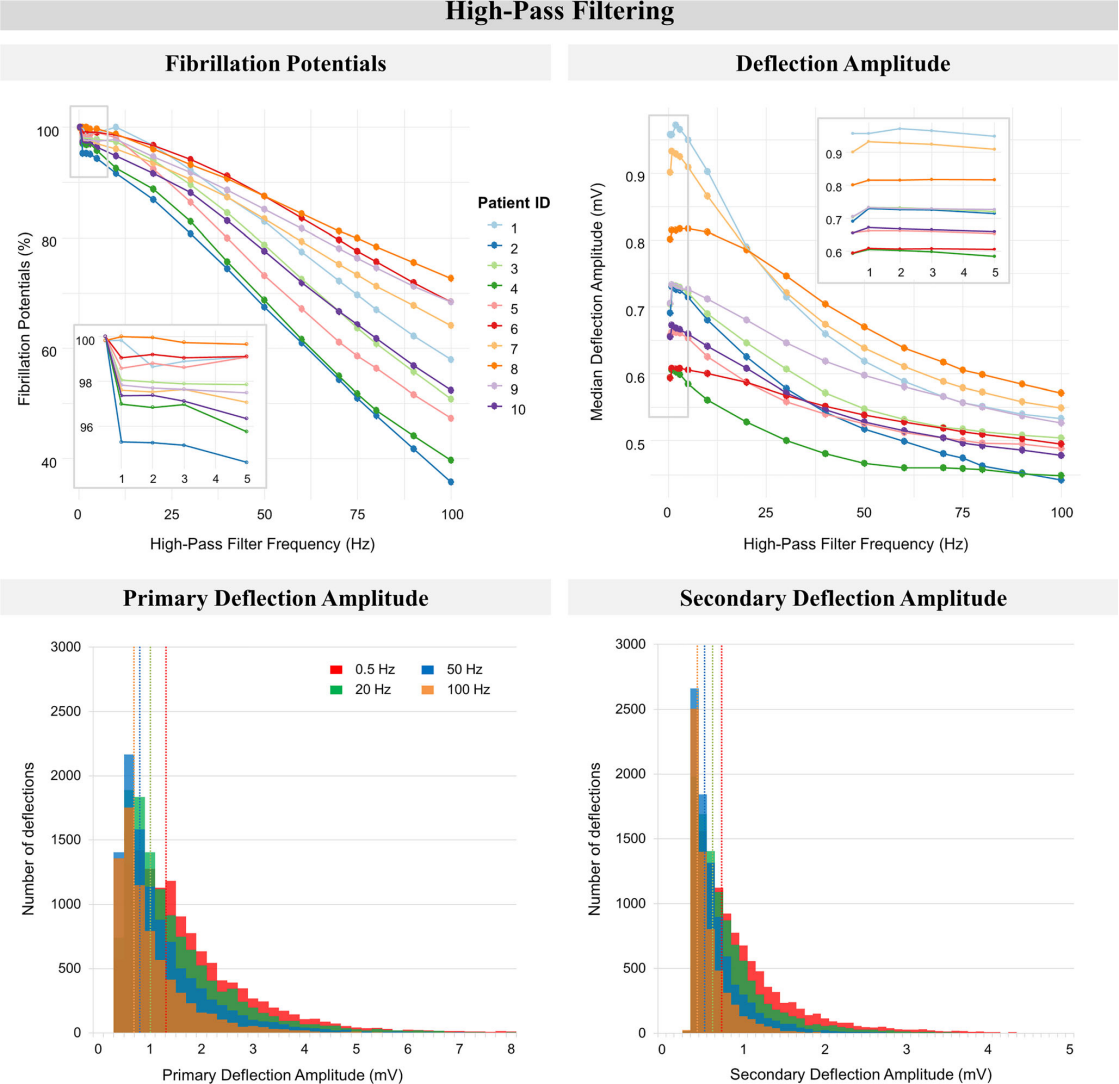
The impact of high-pass filtering on detection of fibrillation potentials and deflection amplitude. *Upper left*: the number of detected fibrillation potentials, expressed as a percentage of maximal number of fibrillation potentials within the patient, of all patients. *Upper right*: overall median deflection amplitude of all patients. *Lower left*: stacked bar-plots of median primary deflection amplitude of one patient. *Lower right*: stacked bar-plots of median secondary deflection amplitude of one patient. For both lower figures, the data of patient 1 was taken as a representative case for all patients. The dotted vertical lines represent the median value of the corresponding stacked bar-plot, representing high-pass filtering at 0.5, 20, 50, or 100 Hz

Increasing the high-pass filter frequency had a negative impact on the number of detected fibrillation potentials and deflection amplitudes, as illustrated in Fig. 3. In the entire study population, the percentage of detected fibrillation potentials gradually decreased, with a loss ranging from 27.2 to 74.5% at the maximum high-pass frequency of 100 Hz (*p* < 0.01, left upper panel). The overall median deflection amplitude decreased with increasing high-pass filtering for all patients (from 0.59–0.96 mV to 0.44–0.57 mV, *p* < 0.01, right upper panel). This negative impact was also observed for median primary and secondary deflection amplitudes separately (*p* < 0.01), and was primarily caused by loss of high amplitude deflections, as illustrated in the histograms in the lower panel of Fig. 3 (obtained from one representative patient).

**Fig. 4:**
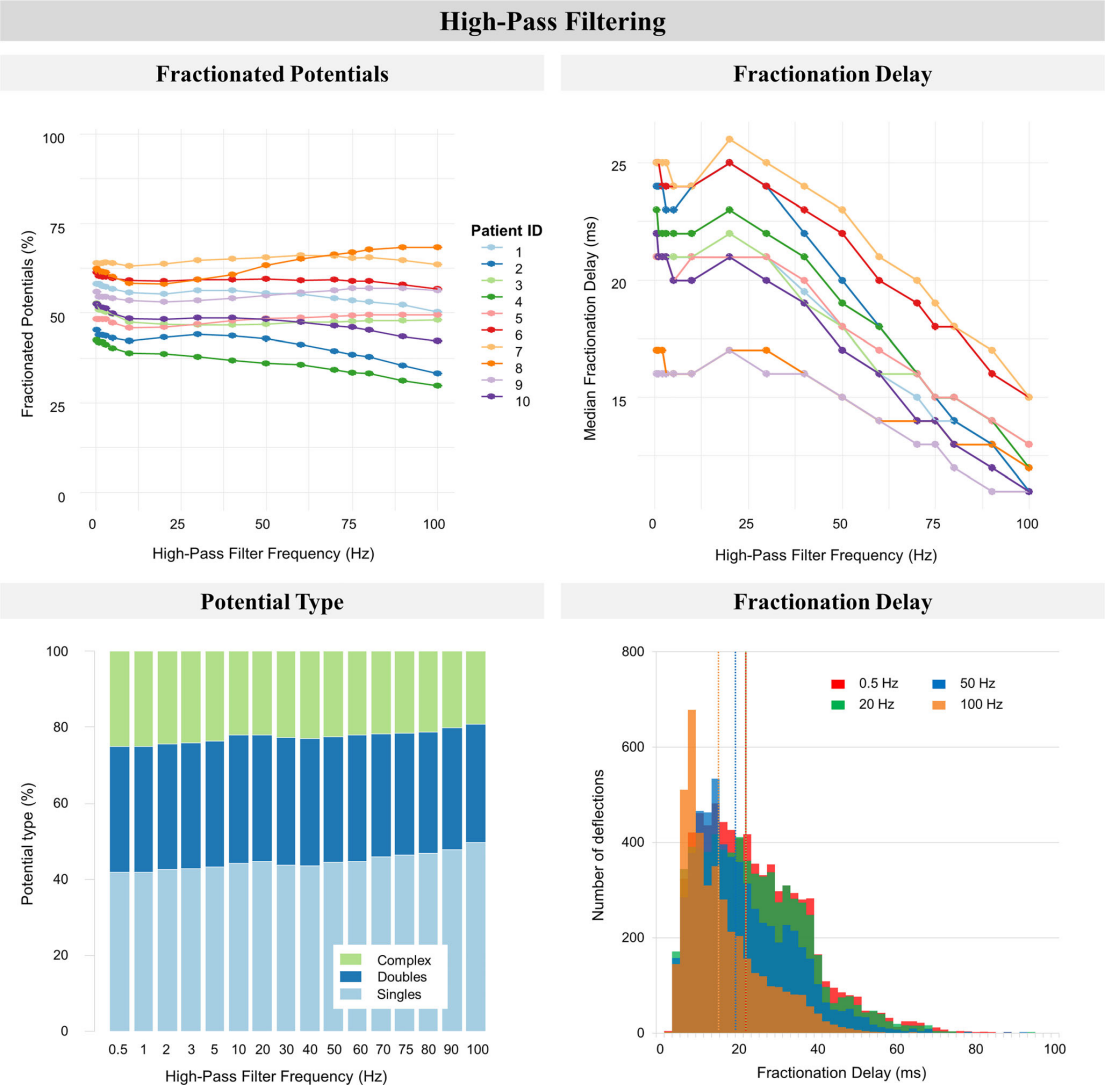
The impact of high-pass filtering on fractionation. *Upper left*: the percentage fractionated potentials (two or more deflections per potential, expressed as a percentage of total detected fibrillation potentials) of all patients. *Upper righ***t**: fractionation delay time (FDT, time interval between first and last deflection) of all patients. *Lower left*: stacked bar-plots of potential types obtained from one patient. Potential type is either single (one deflection), double (two deflections), or complex (three or more deflections) and expressed as a percentage of the total number of detected fibrillation potentials. *Lower right*: stacked bar-plots of median FDT obtained from one patient. The dotted vertical lines represent the median value of the corresponding stacked bar-plot representing high-pass filtering at 0.5, 20, 50, or 100 Hz. For both lower figures, the data of patient 1 was taken as a representative case for all patients

The impact of high-pass filtering on the degree of fractionation, i.e., FDT and distribution of the different fibrillation potential types, is illustrated in Fig. 4. As observed in the left panel of Fig. 4, increasing the high-pass filter frequency from 0.5 to 100 Hz resulted in a slightly increasing percentage of SPs (from 36.1–57.6% to 31.6–70.2%) at the cost of (primarily) CFPs (from 15.9–36.0% to 7.3–37.8%), whereas only a minimal decline in the percentage of DPs was observed (from 25.8–32.9% to 22.5–31.6%) in all patients (all *p* < 0.01). High-pass filtering decreased median FDT for all patients (from 16.0–25.0 ms to 11.0–15.0 ms, right upper panel), primarily due to a loss of fractionated potentials with a long FDT (right lower panel, histogram of one representative patient).

### Impact of Low-Pass Filtering on Morphology of Fibrillation Potentials

**Fig. 5:**
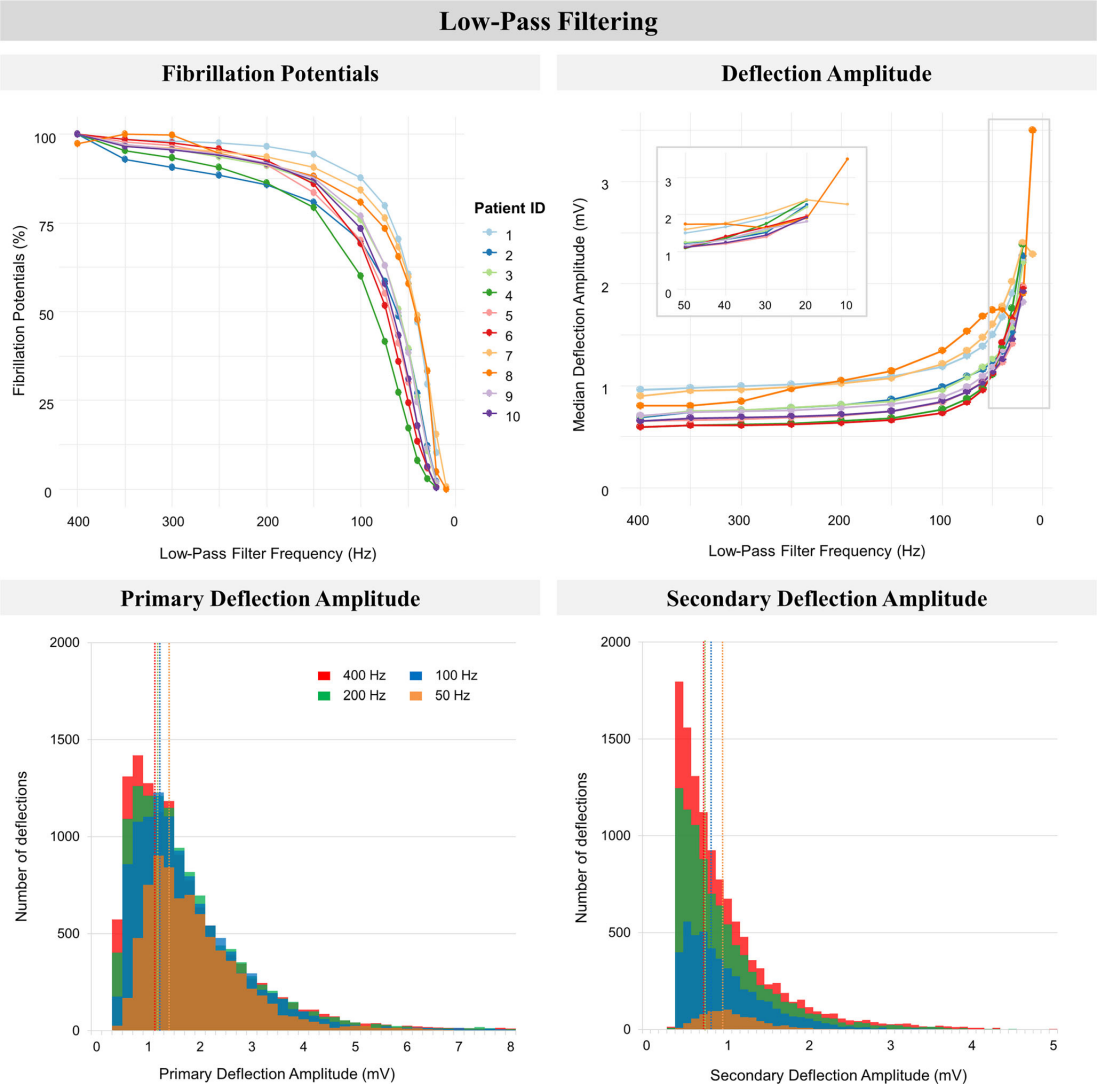
The impact of low-pass filtering on detection of fibrillation potentials and deflection amplitude. *Upper left*: the number of detected fibrillation potentials (expressed as a percentage of maximal number of fibrillation potentials within the patient) of all patients. *Upper right*: overall median deflection amplitude of all patients. *Lower left*: stacked bar-plots of median primary deflection amplitude of one patient. *Lower right*: stacked bar-plots of median secondary deflection amplitude of one patient. For both lower figures, the data of patient 1 was taken as a representative case for all patients. The dotted vertical lines represent the median value of the corresponding stacked bar-plot, representing low-pass filtering at 400, 200, 100 or 50 Hz

**Fig. 6:**
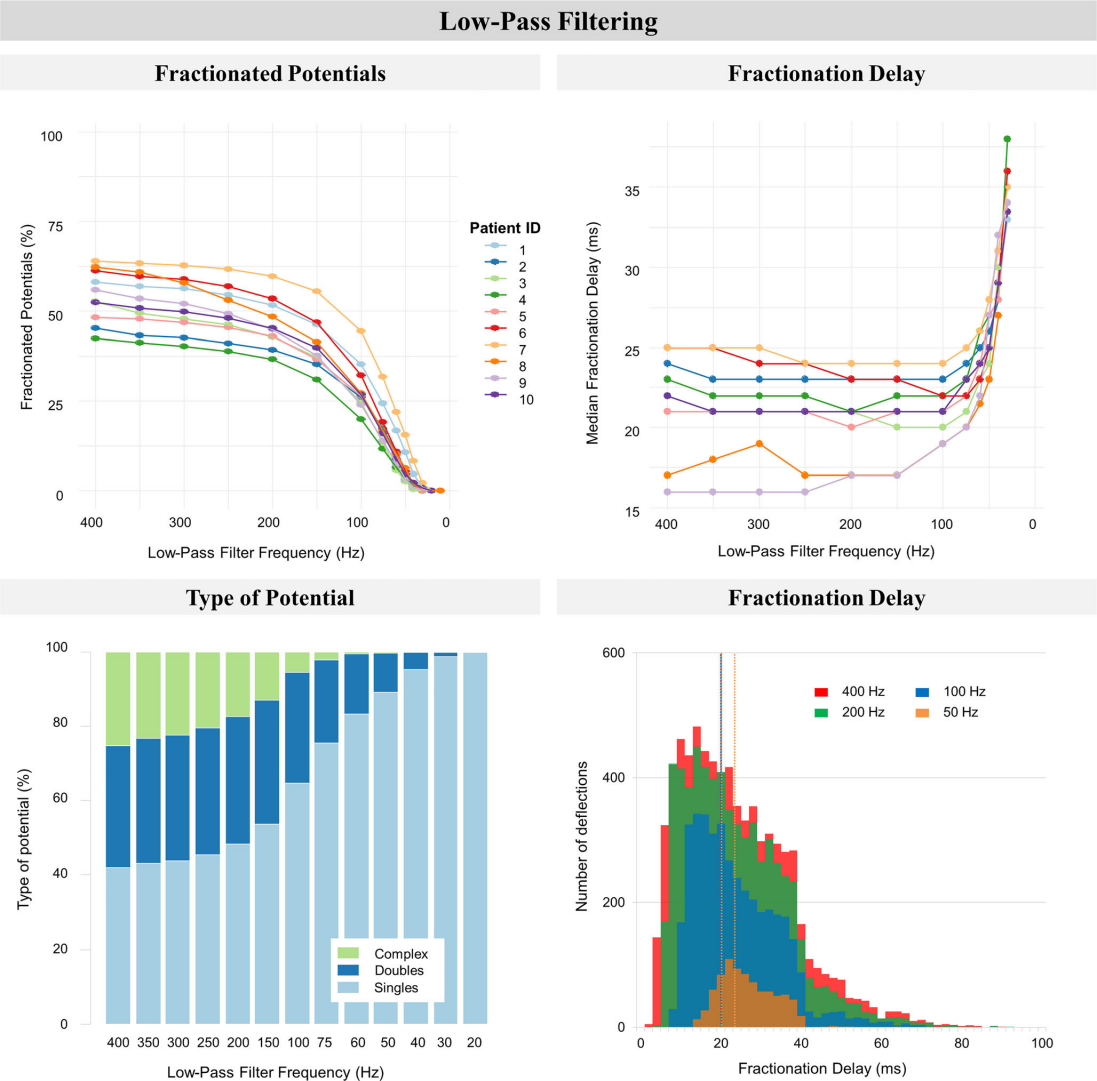
The impact of low-pass filtering on fractionation. *Upper left*: the percentage fractionated potentials (two or more deflections per potential, expressed as a percentage of total detected fibrillation potentials) of all patients. *Upper right*: fractionation delay time (FDT, time interval between first and last deflection) of all patients. *Lower left*: stacked bar-plots with the distribution of potential types of one patient. Potential type is either single (one deflection), double (two deflections), or complex (three or more deflections). Distribution is expressed as a percentage of the total detected fibrillation potentials. *Lower right*: stacked bar-plots of median FDT of one patient. The dotted vertical lines represent the median value of the corresponding stacked bar-plot representing low-pass filtering at 400, 200, 100, or 50 Hz. For both lower figures, the data of patient 1 was taken as a representative case for all patients

The impact of decreasing the low-pass filter frequency on morphology of unipolar fibrillation potentials is shown in Figs. 5 and 6. The left upper panel of Fig. 5 indicates an exponentially decreasing percentage of detected fibrillation potentials with decreasing the low-pass filter frequency (*p* < 0.01). As examples, low-pass filtering at 250 Hz induced a 2.5–11.6% loss of fibrillation potentials, which was 5.6–20.7% at 150 Hz and 20.3–58.4% at 75 Hz. Decreasing the low-pass filter frequency exponentially increased the median deflection amplitude for all patients (from 0.59–0.96 mV to 1.82–2.40 mV, right upper panel), a trend that was also observed within primary and secondary deflections separately. This increase in deflection amplitude is primarily caused by a loss of low-amplitude deflections, as observed in the lower panel (histograms of one representative patient).

In Fig. 6, the impact of low-pass filtering on fractionation is visualized. The left upper panel illustrates a rapid decline of the percentage fractionated potentials (*p* < 0.01). Decreasing the low-pass filter frequency increased the percentage of SPs (from 36.1–57.6% to 100%) at the cost of both DPs (from 25.8–32.9% to 0%) and CFPs (from 15.9–36.0% to 0%). For all patients, the impact of low-pass filtering on DPs was mainly when filtering at 150 Hz or lower, whereas the presence of CFPs diminished almost linearly at frequencies below 400 Hz until none were left (left lower panel, stacked bar-plot of one representative patient). Decreasing the low-pass filter frequency from 400 to 100 Hz did not substantially increase median FDT (from 16.0–25.0 ms to 19.0–24.0 ms respectively, right upper panel), since both fractionated potentials with a short and long FDT disappeared due to the filtering (right lower panel). Low-pass filtering below 100 Hz however resulted in a steep rise of FDT (up to 38.0 ms at 30 Hz), due to the exponential loss of fractionated potentials (right lower panel). The relation between FDT and low-pass filtering was significant (*p* < 0.01).

### Impact of Notch Filtering on Morphology of Fibrillation Potentials

As indicated in Table 2, notch filtering slightly decreased deflection amplitude and increased the percentage of CFPs, whereas no effect on FDT and the percentage of SPs and DPs was observed. Applying a notch filter at 50 Hz induced a loss of ~ 1% detected fibrillation potentials (from 9234–14,545 to 9234–14,405 potentials, *p* = 0.01). This significant, yet minimal, effect was also observed in a decrease in median deflection amplitude (from 0.59–0.96 mV to 0.59–0.95 mV, p < 0.01). Median FDT was not affected by notch filtering (from 16.0–25.0 ms to 16.0–25.0 ms, p = NS). Notch filtering also had no effect on the percentage of SPs and DPs (from 36.1–57.6% to 35.8–57.6% and from 25.8–32.9% to 25.8–32.3% respectively, *p* = NS), but did increase the percentage of CFPs significantly (from 15.9–36.0% to 16.2–37.0%, *p* = 0.0157).

**Table 2:** Impact of notch filtering on unipolar fibrillation potential morphology. Delta values (**∆** = notch filter on–notch filter off) for all parameters and corresponding *p* values based on the likelihood ratio test are given (* < 0.05). Potential type is either single (one deflection), double (two deflections), or complex (three or more deflections). *FDT* fractionation delay time

Study ID	∆ Number of detected fibrillation potentials	∆ Median deflection amplitude (mV)	∆ Singles (%)	∆ Doubles (%)	∆ Complex (%)	∆ Median FDT (ms)
1	− 42	− 0.0125	0.2728	− 0.5923	0.3196	0
2	− 76	− 0.0131	0.1949	− 0.4636	0.2686	0
3	− 132	− 0.0094	0.2474	− 0.1654	− 0.0820	0
4	0	0.0000	0.0000	0.0000	0.0000	0
5	− 213	− 0.0081	− 0.8585	0.4505	0.4080	0
6	− 140	− 0.0050	− 0.3731	0.0758	0.2973	0
7	− 61	− 0.0119	− 0.3039	− 0.6978	1.0017	0
8	14	− 0.0163	− 0.4014	0.2530	0.1484	− 1
9	− 11	− 0.0119	0.3255	− 0.4528	0.1100	0
10	− 190	− 0.0081	− 0.1262	0.0469	0.0875	0
Min–max	− 213–14	− 0.0163–0.0000	− 0.8585–0.3255	− 0.6978–0.4505	− 0.082–1.0017	− 1–0
*p* value	0.0043*	< 0.0001*	0.3827	0.1973	0.0157*	0.3047

### Filtering and Detection of Local Activation Time

As a subanalysis, the impact of filtering on detection of LAT was determined. Results are described in detail in the ESM 3. All filter settings, i.e., all high-pass, low-pass, and notch filter settings, evoked changes in LAT timing (ESM 2). Especially with more aggressive low-pass filtering, the percentage of fibrillation potentials that had a shift in LAT was high (e.g., 58.32 to 63.12% at 100 Hz). Filtering impacted LATs of all potential types (i.e., SPs, DPs, and CFPs). Nevertheless, more complex and long fractionated potentials had a greater ∆LAT—and thus shifted more—than potentials with simpler morphology.

## Discussion

All clinically used mapping systems—both unipolar and bipolar—standardly use signal filters while ablating. Although all mapping systems have different default filter settings, operators have the freedom and ability to change the filter settings according to their wishes. The results of our study clearly show that filtering choices have a significant impact on unipolar signal morphology. Attempts to correct for noise or baseline drift can therefore easily result in erroneous (under)detection of fractionation and/or low-voltage areas and thus ablative targets during mapping.

Our study thereby complements and verifies previously reported findings by Schneider et al. [21] and Lin et al. [22], in which unipolar endocardial peak-to-peak voltage decreased with increasing high-pass filtering in patients with ectopic atrial tachyarrhythmias and atrial flutter, respectively.

### Current Clinical Use of Filtering in Ablative Techniques

Although initially developed as a stand-alone strategy, ablation of tissue exhibiting CFAEs is nowadays generally used as adjuvant therapy to PV isolation, particularly in persistent AF patients [2, 23]. Pathophysiological mechanisms of CFAE include pivoting points, inhomogeneous conduction, functional conduction block, reduced cell coupling, and interstitial fibrosis [2, 24]. On signal level, fractionation is often considered high-frequency content with a low-amplitude [25, 26]. Accordingly, lowering particularly the *low-pass* filter frequency impacted the presence of (complex) fractionated potentials in our study, since high-frequency content was eliminated. So while the decision to change the low-pass filter setting during CFAE ablation is probably made to reject high-frequency noise, detection of CFPs and thereby ablative targets is also strongly impeded. As an example, low-pass filtering at 100 Hz already eliminated up to 40% of fibrillation potentials and reduced the presence of CFPs from 15.9–36.0% to 2.3–10.7% in all patients. Interestingly, increasing *high-pass* filtering did significantly decrease the percentage of fractionated potentials, primarily due to loss of complex fractionated potentials, as also observed in the decreasing FDT. In comparison to the impact of low-pass filtering however, this effect is clearly less substantial. On the contrary, *notch* filtering increased the presence of CFPs (max of 1.0017% increase) by adding artificial components to the unipolar fibrillation potential, just as in bipolar measurements [3].

Our results also stress the significance of adequate (i.e., as minimal as possible) filtering for voltage mapping, as unipolar potential amplitude was impacted by high-pass, low-pass, and notch filtering. Low-voltage areas (< 0.5 mV) have been linked to fibrosis, poor cell-to-cell coupling, slowed and discontinuous electrical conduction, and thus maintenance of AF, motivating targeted ablation of these areas as an isolated approach or in addition to CFAE ablation and/or PV isolation [26, 27]. As our results indicate, *low-pass* filtering decreased the number of low-amplitude deflections, thereby reducing the number of potential target sites for low-voltage ablation in clinical practice. On the contrary, *high-pass* filtering attenuated the overall deflection amplitude and induced an increase in the number of low-amplitude signals, potentially leading to erroneous overdetection of low-voltage target sites. *Notch* filtering did significantly lower deflection amplitude, but its impact is rather small (− 0.0163–0.0000 mV change in amplitude).

With increasing filtering (i.e., increasing high-pass or lowering low-pass cut-off frequencies), the number of detected fibrillation potentials declined. In addition, as described in the Supplemental material, filtering impacted timing of LAT. Though not specifically analyzed in this study, the missing potentials as well as the shift in LATs could lead to changes in detected activation patterns during AF. As such, inadequate filtering could result in unintentional under- or overestimation of rotational activity and focal, or peripheral, waves during mapping. A future study on the precise impact of filtering on measured activation sequences during AF could be very insightful.

As implied in the word itself, filtering inherently results in loss of information. Nevertheless, filtering does not have to be inaccurate if the lost information is irrelevant to your case. The challenge always lies in balancing minimal filtering with maximal signal quality (e.g., without artifacts and noise). However, since the relevant frequency content of fractionated fibrillation potentials is unknown and physiological discrimination between true fractionation and noise contribution is (yet) unfeasible, this study validates the use of minimal filtering. A safer alternative to filtering could be implementation of a signal-to-noise ratio (SNR) within mapping systems, in which detection criteria for fractionated and/or low-voltage potentials become stricter in case of considerable noise, without affecting original signal morphology. Using such an approach also prevents artifacts induced by filtering, such as ringing artifacts, to manifest.

Due to its ability to reduce far-field potentials by subtracting two unipolar electrograms at adjacent sites, bipolar electrograms are clinically often preferred above unipolar measurements. [11] For purposes of fractionation analyses, this favor is perhaps undeserved, since apart from filtering bipolar electrogram morphology is also susceptible to changes in interelectrode distance, electrode size and wavefront direction [3, 11, 12, 28]

### The Pathophysiology of Fractionation

The search for true—pathologic—fractionation remains an ongoing challenge. Differentiating between physiologic fractionation, due to, e.g., overlaying myocardial fibers and functional anisotropy, and pathologic fractionation for now remains difficult. The fact that measurement settings, such as filtering, affect fractionation as well provides another challenge. Nevertheless, the potential of fractionation-guided ablation could be significant if one could find methods for discerning true fractionation identifying abnormal conduction and arrhythmogenicity in AF patients. Specifically high-resolution AF mapping studies, combined with imaging and/or histologic techniques, could aid in unraveling the “fact or artifact” of fractionation.

### Limitations

In this observational study, we included ten patients in whom we measured the impact of high-pass, low-pass, and notch filtering. Even though sample size seems rather small, a total of 3000 s of AF recordings, consisting of 2,557,045 fibrillation potentials were analyzed. Furthermore, our results indicate the impact of filtering to be very much alike between patients. Considering that filtering is a highly reproducible technique, we hypothesize that the general conclusions of this study can be extrapolated to each individual patient. Nevertheless, in clinical practice, there may be variations in filter properties. For example, to assure generalizability and reproducibility we used zero-phase filtering, but this might not be possible in daily clinical practice. For this study, we used unipolar intra-operative high-resolution mapping data, which is different from (bipolar) endocardial mapping data during ablative therapy with typically lower resolution data. For establishing the impact of filtering on unipolar data however, not spatial resolution but temporal resolution—thus sampling rate—is an important and relevant property, since filtering is done in time-domain. Clinically used mapping systems have comparable sampling rates of ~ 1 or 1.2 kHz, so our results should be considered relevant for endocardial unipolar mapping as well. Increasing sampling rate and resolution of analog to digital conversion and using wider band-pass filtering of origin could potentially further improve signal quality.

## Conclusions

High-pass, low-pass, and notch filtering impacted morphology of unipolar fibrillation potentials, including amplitude, fractionation, and FDT, and decreased the number of detected fibrillation potentials, becoming a potential source of error in identification of low-voltage areas and (complex) fractionated potentials. While searching for ablative targets during clinical mapping, operators should be well aware of the consequences of filtering. In case of considerable noise, application of a signal-to-noise ratio—not affecting original signal morphology—could be a safer alternative.

## Electronic supplementary material

ESM 1 Bode plots of the used filters. **Upper panel**: filter frequency response of IIR Butterworth high-pass filter, 2nd-order with 12 dB/octave roll-off with an exemplary half amplitude (−3 dB) cut-off frequency of 50 Hz. **Middle panel**: filter frequency response of IIR Butterworth low-pass filter, 2nd -order with 12 dB/octave roll-off with an exemplary half amplitude (−3 dB) cut-off frequency of 300 Hz. **Lower panel**: filter frequency response of IIR notch filter at 50 Hz, with a quality factor of 30 (PNG 449 kb)

High Resolution Image (TIF 3591 kb)

ESM 2 Impact of high-pass (left panel) and low-pass (right panel) filtering on detection of local activation time (LAT). **Upper left**: effect of high-pass filtering on median ∆LAT (difference in LAT between each filter setting and the default setting) of all patients. **Upper right**: effect of low-pass filtering on median ∆LAT of all patients. **Lower left:** effect of high-pass filtering on number of shifted potentials (i.e., potentials in whom LAT changed, so ∆LAT ≥ 1; expressed as a percentage of number of detected fibrillation potentials at default setting). **Lower right**: effect of low-pass filtering on number of shifted potentials. In the left upper panel, one outlier is not visualized (patient 1: median ∆LAT 17.5 ms at 1 Hz) (PNG 1777 kb)

High Resolution Image (TIF 356 kb)

ESM 3 (DOCX 13 kb)

## Abbreviations

AF: Atrial fibrillation
BB: Bachmann’s Bundle
CFAE: Complex fractionated atrial electrogram
CFP: Complex fractionated potential
DP: Double potential
FDT: Fractionation delay time
LA: Left atrium
LAT: Local activation time
PV: Pulmonary vein
PVL: Left pulmonary vein
PVR: Right pulmonary vein
RA: Right atrium
SNR: Signal-to-noise ratio
SP: Single potential

## References

[1] Konings KT, Smeets JL, Penn OC, Wellens HJ, Allessie MA (1997). Configuration of unipolar atrial electrograms during electrically induced atrial fibrillation in humans. Circulation.

[2] Nademanee K, McKenzie J, Kosar E, Schwab M, Sunsaneewitayakul B, Vasavakul T (2004). A new approach for catheter ablation of atrial fibrillation: Mapping of the electrophysiologic substrate. Journal of the American College of Cardiology.

[3] de Bakker JM, Wittkampf FH (2010). The pathophysiologic basis of fractionated and complex electrograms and the impact of recording techniques on their detection and interpretation. Circulation. Arrhythmia and Electrophysiology.

[4] van der Does LJ, de Groot NM (2017). Inhomogeneity and complexity in defining fractionated electrograms. Heart Rhythm.

[5] Waxman HL, Sung RJ (1980). Significance of fragmented ventricular electrograms observed using intracardiac recording techniques in man. Circulation.

[6] Klitzner TS, Stevenson WG (1990). Effects of filtering on right ventricular electrograms recorded from endocardial catheters in humans. Pacing and Clinical Electrophysiology.

[7] Verma A, Jiang CY, Betts TR, Chen J, Deisenhofer I, Mantovan R (2015). Approaches to catheter ablation for persistent atrial fibrillation. The New England Journal of Medicine.

[8] Providencia R, Lambiase PD, Srinivasan N, Ganesh Babu G, Bronis K, Ahsan S (2015). Is there still a role for complex fractionated atrial Electrogram ablation in addition to pulmonary vein isolation in patients with paroxysmal and persistent atrial fibrillation? Meta-analysis of 1415 patients. Circulation. Arrhythmia and Electrophysiology.

[9] Vogler J, Willems S, Sultan A, Schreiber D, Luker J, Servatius H (2015). Pulmonary vein isolation versus defragmentation: The CHASE-AF clinical trial. Journal of the American College of Cardiology.

[10] Seitz J, Bars C, Theodore G, Beurtheret S, Lellouche N, Bremondy M (2017). AF ablation guided by spatiotemporal Electrogram dispersion without pulmonary vein isolation: A wholly patient-tailored approach. Journal of the American College of Cardiology.

[11] Venkatachalam KL, Herbrandson JE, Asirvatham SJ (2011). Signals and signal processing for the electrophysiologist: Part II: Signal processing and artifact. Circulation. Arrhythmia and Electrophysiology.

[12] Zaman JAB, Schricker A, Lalani GG, Trikha R, Krummen DE, Narayan SM (2014). Focal impulse and rotor mapping (FIRM): Conceptualizing and treating atrial fibrillation. Journal of Atrial Fibrillation.

[13] Grace A, Willems S, Meyer C, Verma A, Heck P, Zhu M (2019). High-resolution noncontact charge-density mapping of endocardial activation. JCI Insight.

[14] van der Does, L. J. M. E., Knops, P., Teuwen, C. P., Serban, C., Starreveld, R., Lanters, E. A. H., et al. (2018). Unipolar atrial electrogram morphology from an epicardial and endocardial perspective. *Heart Rhythm, 15*(6), 879–887. 10.1016/j.hrthm.2018.02.020. 10.1016/j.hrthm.2018.02.020 29476825

[15] Lanters EA, van Marion DM, Kik C, Steen H, Bogers AJ, Allessie MA (2015). HALT & REVERSE: Hsf1 activators lower cardiomyocyt damage; towards a novel approach to REVERSE atrial fibrillation. Journal of Translational Medicine.

[16] van der Does LJ, Yaksh A, Kik C, Knops P, Lanters EA, Teuwen CP (2016). QUest for the Arrhythmogenic substrate of atrial fibRillation in patients undergoing cardiac surgery (QUASAR study): Rationale and design. Journal of Cardiovascular Translational Research.

[17] Teuwen CP, Yaksh A, Lanters EA, Kik C, van der Does LJ, Knops P (2016). Relevance of conduction disorders in Bachmann's bundle during sinus rhythm in humans. Circulation. Arrhythmia and Electrophysiology.

[18] Mouws E, Lanters EAH, Teuwen CP, van der Does L, Kik C, Knops P (2017). Epicardial breakthrough waves during sinus rhythm: Depiction of the Arrhythmogenic substrate?. Circulation. Arrhythmia and Electrophysiology.

[19] Kik C, Mouws E, Bogers A, de Groot NMS (2017). Intra-operative mapping of the atria: The first step towards individualization of atrial fibrillation therapy?. Expert Review of Cardiovascular Therapy.

[20] de Groot NM, Houben RP, Smeets JL, Boersma E, Schotten U, Schalij MJ (2010). Electropathological substrate of longstanding persistent atrial fibrillation in patients with structural heart disease: Epicardial breakthrough. Circulation.

[21] Schneider MA, Ndrepepa G, Weber S, Deisenhofer I, Schomig A, Schmitt C (2004). Influence of high-pass filtering on noncontact mapping and ablation of atrial tachycardias. Pacing and Clinical Electrophysiology.

[22] Lin YJ, Tai CT, Lo LW, Udyavar AR, Chang SL, Wongcharoen W (2007). Optimal electrogram voltage recording technique for detecting the acute ablative tissue injury in the human right atrium. Journal of Cardiovascular Electrophysiology.

[23] Wu SH, Jiang WF, Gu J, Zhao L, Wang YL, Liu YG (2013). Benefits and risks of additional ablation of complex fractionated atrial electrograms for patients with atrial fibrillation: A systematic review and meta-analysis. International Journal of Cardiology.

[24] Konings KT, Kirchhof CJ, Smeets JR, Wellens HJ, Penn OC, Allessie MA (1994). High-density mapping of electrically induced atrial fibrillation in humans. Circulation.

[25] Stiles MK, Brooks AG, Kuklik P, John B, Dimitri H, Lau DH (2008). High-density mapping of atrial fibrillation in humans: Relationship between high-frequency activation and electrogram fractionation. Journal of Cardiovascular Electrophysiology.

[26] Jadidi AS, Lehrmann H, Keyl C, Sorrel J, Markstein V, Minners J (2016). Ablation of persistent atrial fibrillation targeting low-voltage areas with selective activation characteristics. Circulation. Arrhythmia and Electrophysiology.

[27] Blandino A, Bianchi F, Grossi S, Biondi-Zoccai G, Conte MR, Gaido L (2017). Left atrial substrate modification targeting low-voltage areas for catheter ablation of atrial fibrillation: A systematic review and meta-analysis. Pacing and Clinical Electrophysiology.

[28] Stevenson WG, Soejima K (2005). Recording techniques for clinical electrophysiology. Journal of Cardiovascular Electrophysiology.

