# Supplementary figures and images for: The Impact of Filter Settings on Morphology of Unipolar Fibrillation Potentials
Source: J Cardiovasc Transl Res. 2020 May 14;13(6):953–64. doi: 10.1007/s12265-020-10011-w (PMC7708344; doi:10.1007/s12265-020-10011-w)

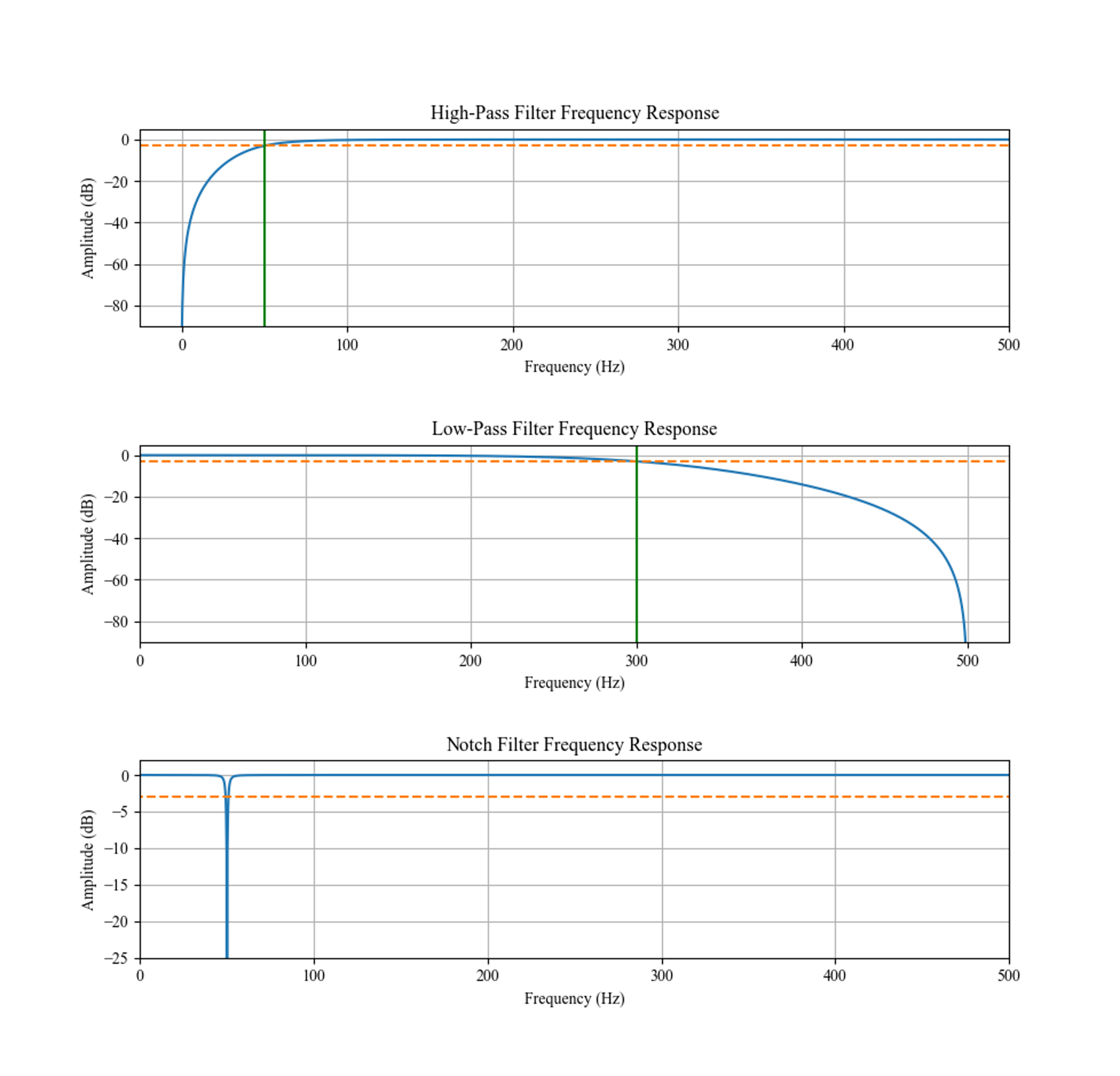

Supplement: Supplementary file 1 — Bode plots of the used filters. Upper panel: filter frequency response of IIR Butterworth high-pass filter, 2nd-order with 12 dB/octave roll-off with an exemplary half amplitude (−3 dB) cut-off frequency of 50 Hz. Middle panel: filter frequency response of IIR Butterworth low-pass filter, 2nd -order with 12 dB/octave roll-off with an exemplary half amplitude (−3 dB) cut-off frequency of 300 Hz. Lower panel: filter frequency response of IIR notch filter at 50 Hz, with a quality factor of 30 (PNG 449 kb) [file 12265_2020_10011_Fig7_ESM.png]

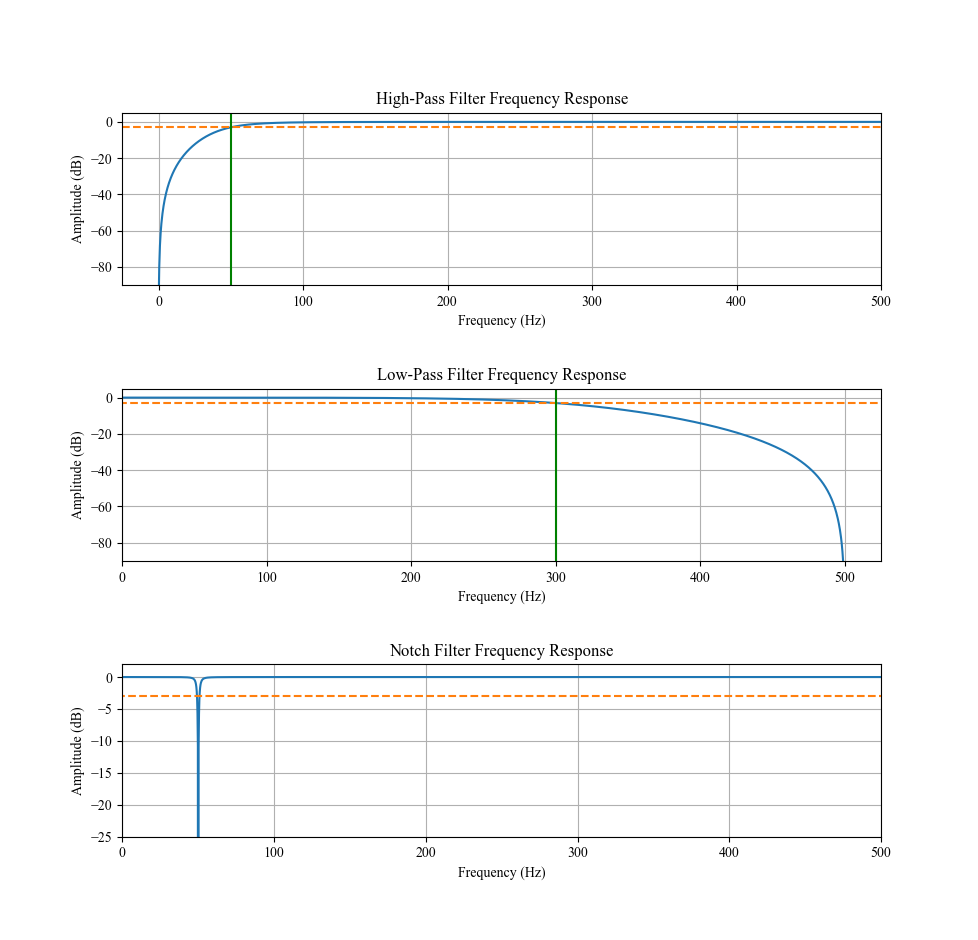

Supplement: Supplementary file 2 — High Resolution Image (TIF 3591 kb) [file 12265_2020_10011_MOESM1_ESM.tif]

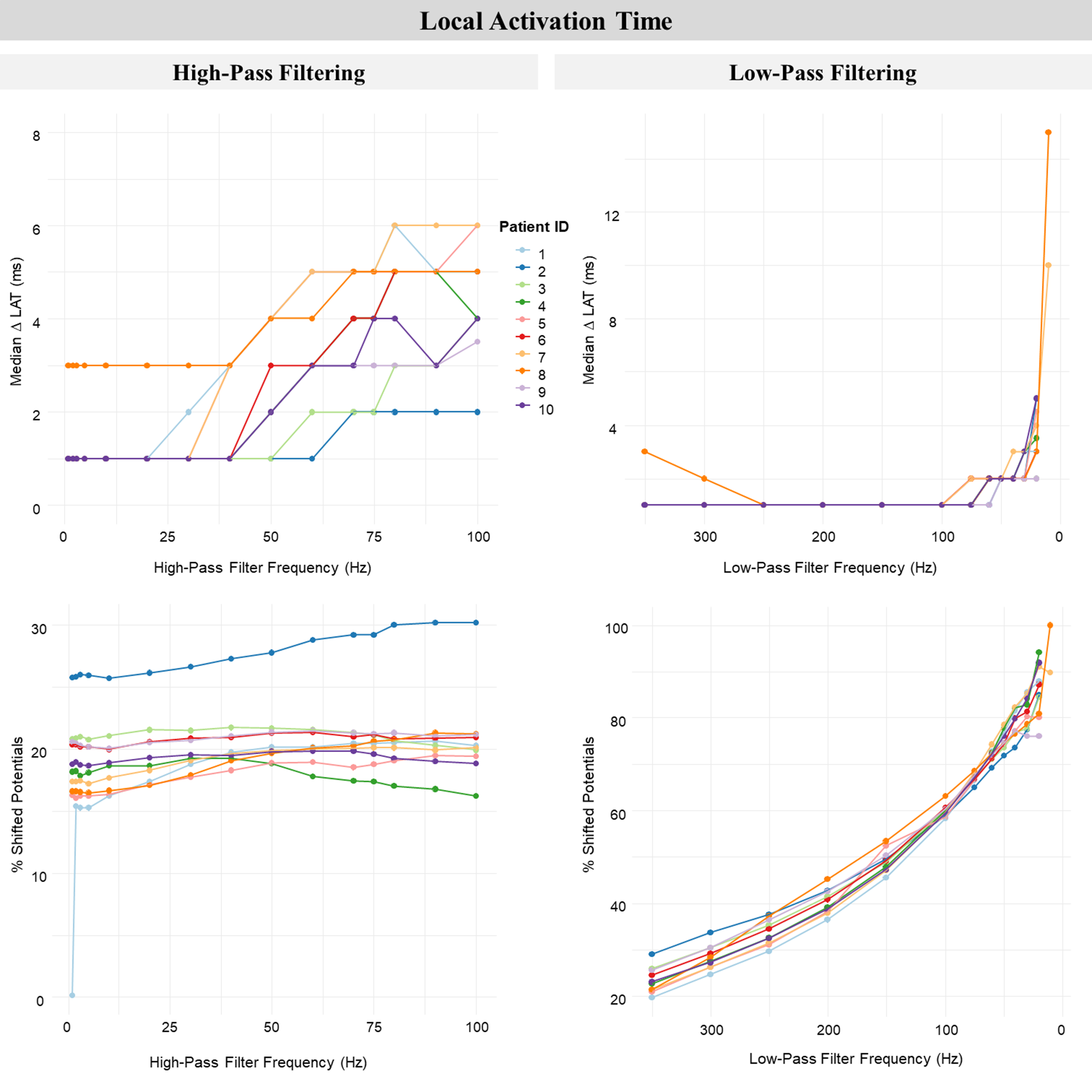

Supplement: Supplementary file 3 — Impact of high-pass (left panel) and low-pass (right panel) filtering on detection of local activation time (LAT). Upper left: effect of high-pass filtering on median ∆LAT (difference in LAT between each filter setting and the default setting) of all patients. Upper right: effect of low-pass filtering on median ∆LAT of all patients. Lower left: effect of high-pass filtering on number of shifted potentials (i.e., potentials in whom LAT changed, so ∆LAT ≥ 1; expressed as a percentage of number of detected fibrillation potentials at default setting). Lower right: effect of low-pass filtering on number of shifted potentials. In the left upper panel, one outlier is not visualized (patient 1: median ∆LAT 17.5 ms at 1 Hz) (PNG 1777 kb) [file 12265_2020_10011_Fig8_ESM.png]

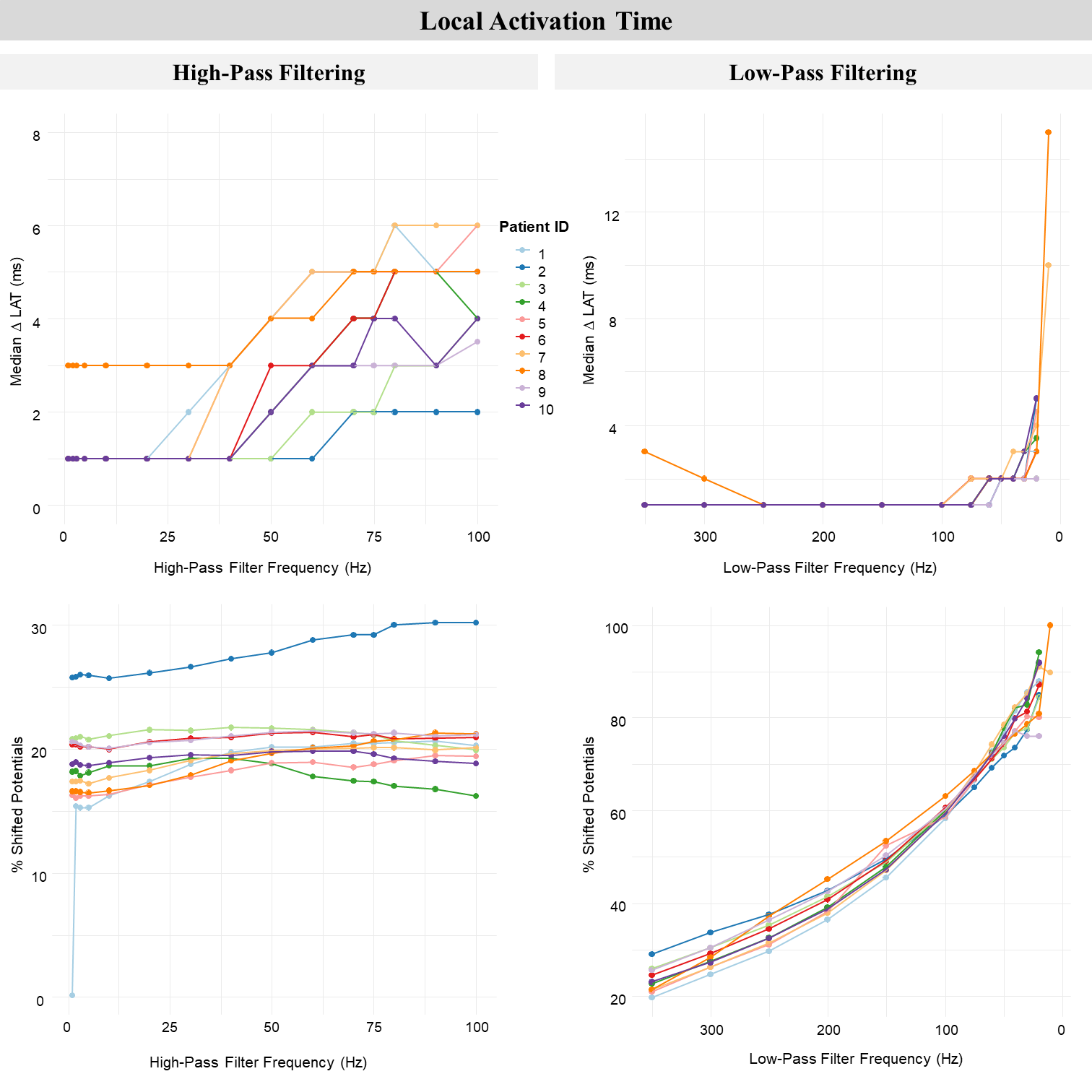

Supplement: Supplementary file 4 — High Resolution Image (TIF 356 kb) [file 12265_2020_10011_MOESM2_ESM.tif]
