# Supplementary material for: The Impact of Filter Settings on Morphology of Unipolar Fibrillation Potentials
Source: J Cardiovasc Transl Res. 2020 May 14;13(6):953–64. doi: 10.1007/s12265-020-10011-w (PMC7708344; doi:10.1007/s12265-020-10011-w)
Supplement: Supplementary file 5 — (DOCX 13 kb) [file 12265_2020_10011_MOESM3_ESM.docx]

**Supplemental material**

**Impact of filtering on detection of local activation time**

As illustrated in *Figure 1,* the steepest negative deflection of a potential was marked as the local activation time (LAT). As a subanalysis, we investigated the impact of additional high-pass, low-pass and notch filtering on timing of LAT. For this purpose, the difference in LAT (∆LAT) between each filter setting (see paragraph *Filter settings*) and the default setting (i.e. 0.5-400 Hz without notch) for all potentials in the 192-electrode array was determined. A median ∆LAT value of all atrial potentials within the 192-array was derived and compared between filter settings.

*High-pass filtering*

Increasing the high-pass filter frequency increased ∆LAT, as illustrated in *Supplemental Figure 2* (p<0.01, upper left panel). At the maximum high-pass frequency of 100 Hz, the median ∆LAT ranged from 2 to 6 ms. With increasing high-pass filtering, the percentage of potentials with a shift in LAT (i.e. ∆LAT ≥ 1) slightly increased (from 0.14-25.75% to 16.24-30.12%, p<0.01, lower left panel). In total, 286664 potentials shifted due to high-pass filtering, of which 33.21% were single potentials (one deflection, SP), 32.31% double potentials (two deflections, DP) and 34.48% complex fractionated potentials (more than two deflections, CFP). Overall, median ∆LAT (p5-p95) was 1 ms (1-21 ms) for SPs, 3 ms (1-30 ms) for DPs and 6 ms (1-31 ms) for CFPs.

*Low-pass filtering*

The impact of decreasing the low-pass filter frequency on timing of LAT is shown in the right panel of *Supplemental Figure 2*. The right upper panel indicates an exponentially increasing ∆LAT with decreasing the low-pass filter frequency (p<0.01, from 1-3 ms to 2-15 ms). For all patients, decreasing the low-pass filter frequency increased the percentage of potentials with a shift in LAT (from 19.62-28.96% to 76.05-94.12%, p<0.01, right left panel). In total, 417119 potentials shifted due to low-pass filtering, of which 33.69% were SPs, 32.29% DPs and 34.02% CFPs. Overall, median ∆LAT (p5-p95) was 1 ms (1-8 ms) for SPs, 1 ms (1-27 ms) for DPs and 4 ms (1-31 ms) for CFPs.

*Notch filtering*

In nine out of ten patients, notch filtering lead to a shift in LAT of fibrillation potentials. In these patients, median ∆LAT ranged from 8 to 10 ms and 3.61 to 6.99% of potentials had a shift in LAT. A total of 5188 potentials shifted due to notch filtering, of which 32.79% were SPs, 37.95% DPs and 29.26% CFPs. Overall, median ∆LAT (p5-p95) was 3 ms (1-30 ms) for SPs, 10 ms (1-33 ms) for DPs and 11 ms (1-33 ms) for CFPs.

Morphological changes evoked by filtering as described in the manuscript, thereby induce the LAT of fibrillation potentials to shift, especially with more aggressive low-pass filtering. Although filtering impacted LATs of all potential types (i.e. SPs, DPs and CFPs), more complex and long fractionated potentials had a greater ∆LAT – and thus shifted more – than potentials with simpler morphology.
